# Albumin Binds COVID-19 Spike 1 Subunit and Predicts In-Hospital Survival of Infected Patients—Possible Alteration by Glucose

**DOI:** 10.3390/jcm11030587

**Published:** 2022-01-25

**Authors:** Khaoula Zekri-Nechar, José J. Zamorano-León, Antonio Segura-Fragoso, José R. Alcaide, Carmen Reche, Alcira Andrés-Castillo, Carlos H. Martínez-Martínez, Manel Giner, Rodrigo Jiménez-García, Ana López-de-Andrés, Carlos Navarro-Cuellar, Miguel A. García-Fernández, Antonio López-Farré

**Affiliations:** 1 Department of Medicine, Faculty of Medicine, Universidad Complutense de Madrid, 28040 Madrid, Spain; kzekri@ucm.es (K.Z.-N.); alciraan@ucm.es (A.A.-C.); carloshugomar@gmail.com (C.H.M.-M.); magfeco@ucm.es (M.A.G.-F.); antonio.lopez.farre@med.ucm.es (A.L.-F.); 2 Department of Public Health and Maternal and Child Health, Faculty of Medicine, Universidad Complutense de Madrid, IdISSC, 28040 Madrid, Spain; rodrijim@ucm.es (R.J.-G.); anailo04@ucm.es (A.L.-d.-A.); 3 School of Health Sciences, Universidad de Castilla La Mancha, 45600 Talavera de la Reina, Spain; asegurafr@gmail.com; 4 Department of Health and Emergency, Community of Madrid Ministry, 28013 Madrid, Spain; joseralcaide2@gmail.com; 5 Emergency Department, Gómez Ulla Central Defense Hospital, 28047 Madrid, Spain; carmen.reche@hotmail.com; 6 Department of Surgery, Faculty of Medicine, Universidad Complutense de Madrid, 28040 Madrid, Spain; manginer@ucm.es; 7 Maxillofacial Surgery Department, Hospital General Universitario Gregorio Marañón, 28007 Madrid, Spain; cnavarrocuellar@gmail.com

**Keywords:** albumin, COVID-19, glucose, glycated albumin, in-hospital mortality, spike protein S1 subunit

## Abstract

(1) Background: This study aimed to analyze if the serum albumin levels of hospitalized SARS-CoV-2 (COVID-19) patients on admission could predict <30 days in-hospital all-cause mortality, and if glucose levels on admission affected this predictive ability. (2) Methods: A multicenter retrospective cohort of 1555 COVID-19-infected adult patients from public hospitals of the Madrid community were analyzed. (3) Results: Logistic regression analysis showed increased mortality for ages higher than 49 y. After adjusting for age, comorbidities and on-admission glucose levels, it was found that on-admission serum albumin ≥3.5 g/dL was significantly associated with reduced mortality (OR 0.48; 95%CI:0.36–0.62). There was an inverse concentration-dependent association between on-admission albumin levels and <30 days in-hospital all-cause mortality. However, when on-admission glucose levels were above 125 mg/dL, higher levels of serum albumin were needed to reach an association with survival. In vitro experiments showed that the spike protein S1 subunit of SARS-CoV-2 binds to native albumin. The binding ability of native albumin to the spike protein S1 subunit was decreased in the presence of an increasing concentration of glycated albumin. (4) Conclusions: On-admission serum albumin levels were inversely associated with <30 days in-hospital all-cause mortality. Native albumin binds the spike protein S1 subunit, suggesting that native albumin may act as a scavenger of the SARS-CoV-2 virus.

## 1. Introduction

Albumin is a protein synthesized in the liver and composed of a single polypeptide chain containing 585 amino acids, 17 disulfide bridges and 3 homologous domains that are connected in a helical structure. Albumin represents around 60% of the total proteins in the blood, being the main blood carrier of molecules such as free fatty acids, metals, medical drugs, and even bacteria and viruses [1,2].

Albumin is considered as a biomarker of both malnutrition and illness severity [3,4]. In fact, inflammatory processes that reduce albumin levels and hypoalbuminemia (serum albumin levels <3.5 g/dL) have been used as a predictor of poor outcomes and high mortality in acute critically ill patients [5].

The SARS-CoV-2 virus (COVID-19) pandemic continues to cause high mortality, even though global vaccination has been accelerated. Additionally, multiple variants of the virus that causes COVID-19 are appearing and, as the pandemic progresses globally, much remains unknown about the disease dynamics and its risk factors [6]. As already mentioned, although albumin has been considered a predictor for hospitalized patients with severe disease, surprisingly, the association between the mortality risk of patients hospitalized for COVID-19 and the function of serum albumin levels has only been evaluated in a very limited way. The reported studies looking at albumin as a predictor of mortality for COVID-19 patients included a very limited number of severe COVID-19 patients [7,8]. In this regard, the limited number of patients followed in these studies and the type of COVID-19 patients included probably limited the statistical power of the observed results and their clinical impact.

Another important factor that may directly affect albumin is glucose. Glucose binds to albumin, promoting albumin glycation through a non-enzymatic spontaneous reaction [9].

Glycation of albumin diminishes the binding of albumin to other molecules [10]. Interestingly, independently of diabetes mellitus, hyperglycemia has emerged as a risk factor for all-cause mortality in non-critically ill hospitalized COVID-19 patients [11].

The aim of the present work was to analyze whether on-admission serum albumin levels may predict the risk of <30 days in-hospital all-cause mortality in a cohort of COVID-19-infected patients who were hospitalized in public hospitals of the Madrid community (Spain), during the second wave of the COVID-19 pandemic. We also evaluated if, independently of diabetes mellitus, plasma glucose levels on admission may modify the association of albumin levels with <30 days all-cause mortality in COVID-19 patients.

Since the results demonstrated that on-admission albumin levels predicted <30 days all-cause mortality in confirmed COVID-19 patients, and that this was affected by on-admission glucose levels, additional in vitro experiments were performed to analyze the possibility that native albumin and/or glycated albumin may bind the SARS-CoV-2 spike S1 and S2 subunits.

## 2. Materials and Methods

### 2.1. Study Population

**Figure 1:**
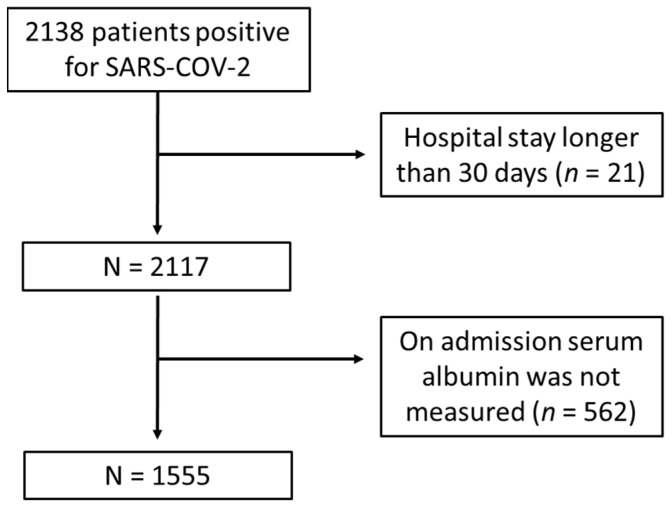
Flow chart of study population inclusion and exclusion criteria. This flow diagram illustrates the resulting number of 1555 recruited patients positive for SARS-CoV-2 admitted for hospitalization in 24 different public hospitals of the community of Madrid (Spain), between August-November 2020; and the number and reasons for excluding COVID-19 patients based our pre-determined criteria. Following this process, 21 patients were removed due to a hospital stay higher than 30 days, while 562 patients were removed because on admission serum albumin was not collected.

Our study was carried out using retrospective data from a completely anonymized database provided by the Madrid Community Ministry of Health. The database included 1555 patients with SARS-CoV-2 infected adult patients confirmed by reverse transcription polymerase chain reaction (RT-PCR) testing of a nasopharyngeal sample, sputum or bronchoalveolar lavage samples. Patients were over 18 years old, and they had been admitted for hospitalization in 24 different public hospitals of the community of Madrid. Data were collected between 17 August and 29 November 2020. Inclusion criteria included a SARS-CoV-2 infection confirmed by RT-PCR and any of the following criteria: respiratory rate ≥30 breaths per minute, and/or finger oxygen saturation ≤93% at rest, and/or PaO_2_/FiO_2_ ≤ 300 mmHg. Serum albumin levels at admission and age above 18 years were also considered as inclusion criteria. Serum albumin concentration was analyzed using automated colorimetric methods based on the bromocresol green method. The range of measurement levels for serum albumin depended on the automatic biochemical analyzer used in each hospital, and ranged between 1.5 and 5.5 g/dL. Hypoalbuminemia was defined as serum albumin concentration below 3.5 g/dL [12,13]. On admission, glycemic level was measured in the central laboratory of each hospital through routine ematobiochemistry tests. Results were measured in mg/dL and grouped according to classification: lower than 100 mg/dL, from 100 to 125 mg/dL, and higher than 125 mg/dL. Exclusion criteria included a hospital stay longer than 30 days. Survival was only considered when patients were discharged home within 30 days after hospital admission. We only included symptomatic patients with a positive COVID-19 diagnosis at admission in the emergency departments of the hospitals. This left us with our final cohort of 1555 patients who met the inclusion criteria (Figure 1). Patients that acquired COVID-19 during hospitalization and/or were asymptomatic were not included in the anonymized database provided by the Madrid Community Ministry of Health. The existence of the comorbidities reflected in the above-mentioned database were included in the analysis.

The primary clinical endpoint was <30 days all-cause mortality during hospitalization.

The analysis was conducted in accordance with the Declaration of Helsinki. This analysis is part of a larger study to develop artificial intelligence algorithms to identify biomarkers of poor prognosis in COVID-19 confirmed patients, and it was approved by our Local Ethics Committee (21/084-E).

### 2.2. Interaction between Native and Glycated Albumin with SARS-CoV-2 Spike Protein Subunits

Native albumin (12667, Sigma-Aldrich, Merck KGaA, Darmastadt, Germany) 0, 1 and 3.5 g/dL were incubated in a humidified incubator containing 5% CO_2_ for 24 h at 37 °C in RPMI medium with a cocktail of recombinant SARS-CoV-2 spike protein subunits S1 and S2 (10 nmol/L each) (SARS-CoV-2 subunit S1, Cat No 230–01101 and SARS-CoV-2 subunit S2 Cat No 230–01103. RayBiotech, Peachtree Corners, GA, USA) derived from *E. coli*. The choice of these concentrations of the spike protein subunits was based on previous studies that tested the in vitro effects of spike proteins on stimulating human immune cells and primary human brain endothelial cells [14,15]. Additional experiments were carried out replacing each native albumin concentration with 25% and 50% glycated albumin (A8301, Sigma, Sigma-Aldrich, St. Louis, MO, USA). Moreover, experiments using the RBD region of the COVID-19 spike protein (RP-87678, Invitrogen, Thermo Fisher Scientific, Waltham, MA, USA) were also carried out.

After coincubation of the spike protein subunits with native albumin and glycated albumin, a monoclonal antibody against human albumin (1:25, MA5-29022 Invitrogen, Thermo Fisher Scientific, Waltham, MA, USA) was added and incubated overnight at 4 °C. Then, protein A (10 µg/mL) was added to each sample, and they were incubated for an additional 2 h. After washing twice and being resuspended in 50µL Laemmli buffer, 20µL of each sample was loaded and developed in 15% SDS/PAGE. After blotting, nitrocellulose membranes were blocked with 5% (*w*/*v*) bovine serum albumin and then incubated with a polyclonal antibody against the S1 spike subunit (1:1000 PA5-81795. Invitrogen, ThermoFisher Scientific, Waltham, MA, USA) and a monoclonal antibody against S2 (1:1000 MAB 10557. R&D Systems, McKinley Place NE, MN, USA) COVID-19 spike subunits. After washing, nitrocellulose membranes were then incubated with a peroxidase-conjugated anti-rabbit IgG for the S1 antibody (1:2500) and an anti-mouse IgG for the S2 antibody (1:2500), developed using chemiluminescence reagents (ECL; GE Healthcare, Little Chalfont, Buckinghamshire, UK) and detected using an Ibright Imaging System (Ibright FL100, ThermoFisher Scientific, Waltham, MA, USA).

### 2.3. Statistical Analysis

The variables were expressed as frequency and percentage. Associations with mortality were analyzed using the Chi-square test. Two groups were randomly performed. The first referral group (*n* = 801) was used to identify the prediction model. Then, prediction model was validated in the remaining patients (*n* = 754). Concordance index (C-index) for right-censored data was applied to evaluate the performance of prediction models. Univariate and multivariate mortality Odds Ratios (OR) were calculated by logistic regression analysis. The 95% confidence interval (CI) and *p*-value for each OR were tabulated. The Hosmer–Lemeshow test was used as statistical test for goodness of fit for the generated regression model. To analyse the binding capability of different concentrations of native and glycated albumins to bind the SARS-CoV-2 spike subunits S1 and S2, the Wilcoxon test was used comparing each specific concentration with the experiments performed in the absence of albumin.

Statistical significance was considered for *p* values < 0.05. The statistical analysis was performed with the SPSS software (version 25.0).

## 3. Results

### 3.1. Demographic Characteristics

As shown in Table 1, most of the COVID-19 patients were older than 50 years. The mean age was 67.61 ± 0.46 years. Gender distribution was similar, although there were slightly more men than women (Table 1). The more frequent comorbidities were hypertension and dyslipidemia, followed by diabetes mellitus (Table 1). The database also contained confirmed COVID-19 patients with active cancer or chronic obstructive pulmonary disease (COPD) (Table 1).

During the <30 days hospitalization follow-up, the incident of venous thromboembolism, including deep venous or pulmonary thromboembolisms, was small (2.8% of all included patients). Only 11.2% of the hospitalized patients required the intensive care unit (ICU). Almost a third of the confirmed COVID-19 patients included in the database died within 30 days of in-hospital follow-up (Table 1).

### 3.2. Associations with In-Hospital All-Cause Mortality

Age was associated with <30 days in-hospital all-cause mortality (Table 2). Mortality seems to increase as COVID-19 patient’s age increases (Table 2). There was no association between gender and mortality (Table 2).

The most frequent comorbidity associated with <30 days all-cause mortality was active cancer followed by COPD (Table 2). Hypertension, dyslipidemia and diabetes mellitus were also likely to be associated with <30 days in-hospital all-cause mortality (Table 2). ICU admission was significantly associated with <30 days in-hospital all-cause mortality (Table 2). On-admission albumin and glucose levels, but not C-reactive protein, were associated with <30 days in-hospital all-cause mortality (Table 2).

Referral (*n* = 801) and validation (*n* = 754) groups were randomly performed to prove that native albumin data can be used to predict outcomes of COVID-19 patients. No significant differences were found in the frequency of the analysed variables among both groups (Supplementary Table S1). The prediction model was performing using a referral group. This model was successfully validated with the validation group (Supplementary Table S2). Concordance index values of 0.836 and 0.879 were obtained for the referral and validation groups, respectively. These results indicated that a deceased subject was correctly classified as deceased by the proposed prediction model with probability higher than 83%. Then, this prediction model was accepted and used with all patients (Table 3).

Through logistic regression analysis mortality OR was assessed for the variables that reached association with <30 days in-hospital all-cause mortality. As shown in Table 3, univariate analysis demonstrated that older age, active cancer and ICU admission were predictors of <30 days all-cause mortality.

The highest mortality OR was found for age ≥85 years and mortality OR values were attenuated with decreasing age (Table 3). Multivariate analysis showed that mortality OR by age was not changed after the adjustment for comorbidities and on-admission levels of albumin and glucose (Table 3). Multivariate analysis also showed active cancer was an independent predictor of <30 days in-hospital all-cause mortality (Table 3).

Univariate mortality OR for dyslipidemia, diabetes and hypertension were statistically significant, although this significance was lost after adjustment for age, gender and all the other variables (Table 3).

Mortality OR was significantly reduced among patients showing on-admission serum albumin ≥3.5 g/dL (Table 3). The predictive value of on-admission serum albumin levels persisted after adjustment for age, gender, comorbidities, ICU admission and on-admission glucose levels (Table 3).

On-admission glucose levels above 125 mg/dL, but not lower glucose levels (100–125 mg/dL), significantly increased mortality OR. This finding remained significant after adjustment for age, gender, comorbidities, ICU admission and on-admission albumin levels (Table 3).

### 3.3. Relationship between On-Admission Serum Albumin Levels and In-Hospital Mortality

**Figure 2:**
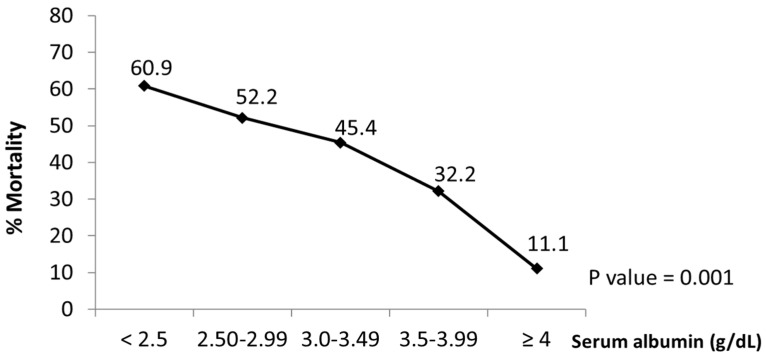
Association between serum albumin and mortality in COVID-19 patients. The graph shows the decreased rates (%) of in-hospital mortality at 30 days from all causes associated with on admission increased serum albumin levels in COVID-19 patients. Data obtained from the multi-center cohort-study on impact of admission serum albumin levels for the prediction of <30 days in-hospital all-cause mortality in COVID-19 patients, Spain, August-November 2020 (n = 1555).

As shown in Figure 2, in the confirmed COVID-19 patients, the <30 days in-hospital all-cause mortality was inversely associated with on-admission albumin levels. In fact, as serum albumin levels increased, the <30 days all-cause in-hospital mortality significantly decreased (Figure 2). Within 30 days after hospital admission, 88.9% of COVID-19 patients were discharged home when on-admission albumin levels were ≥4 g/dL (Figure 2). However, only 39.1% of COVID-19 patients showing on-admission albumin levels < 2.5 g/dL were discharged alive (Figure 2). These percentages were not modified by gender (*p* > 0.05).

**Figure 3:**
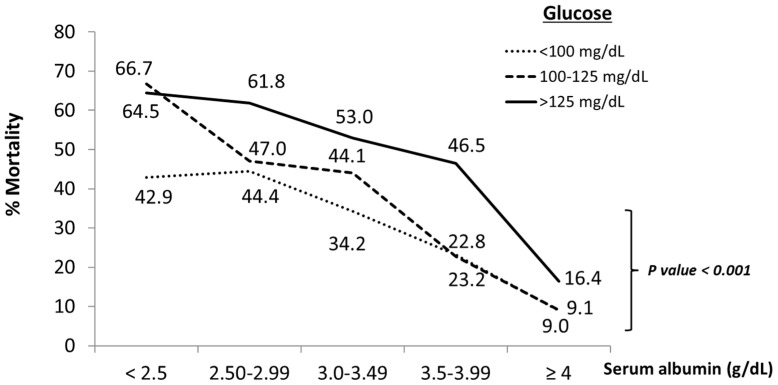
Effect of glucose levels on association between increased serum albumin and decreased mortality rates in COVID-19 patients. The graph shows change in rates (%) of in-hospital mortality at 30 days from all causes associated with increased serum albumin levels by low (<100 mg/dL), normal (100 to 125 mg/dL) and high (>125 mg/dL) glucose levels on admission in COVID-19 patients. Data obtained from the multi-center cohort-study on impact of admission serum albumin levels for the prediction of <30 days in-hospital all-cause mortality in COVID-19 patients, Spain, August–November 2020 (*n* = 1555).

Interestingly, patients with low albumin levels (<3.5 g/dL) and low glucose levels (<100 mg/dL) showed a lower mortality rate than patients with low albumin levels (<3.5 g/dL) but with glucose levels above 100 mg/dL (Figure 3). This observation was more evident when on-admission albumin levels were below 2.5 g/dL (Figure 3). Gender did not modify these results (*p* > 0.05).

Confirmed COVID-19 patients showing on-admission glucose levels above 125 mg/dL showed a significantly increased <30 days in-hospital all-cause mortality for all albumin levels, as compared with patients whose on-admission glucose levels were <125 mg/dL (Figure 3).

### 3.4. Binding of SARS-CoV-2 Spike Subunits to Albumin

**Figure 4:**
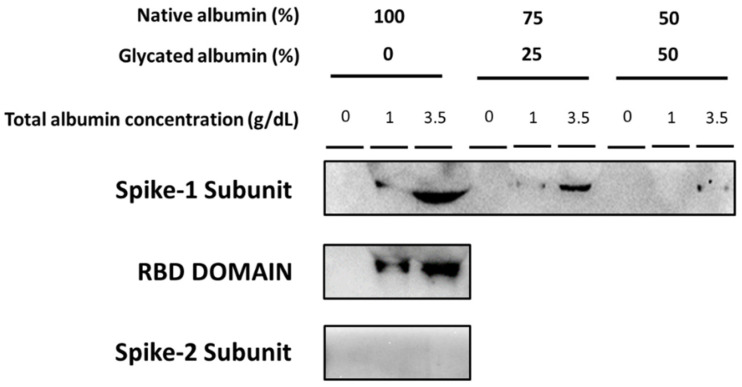
Binding ability of COVID-19 spike subunits to native and glycated albumin in vitro. Representative Western blots show the binding of recombinant SARS-CoV-2 spike protein S1 subunit to 1 g/dL and 3.5 g/dL native albumin. Experiments where each final albumin concentration of native albumin (1 g/dL and 3.5 g/dL) was replaced by glycated albumin (25% and 50%) are also represented. The figure also shows a representative Western blot showing the binding of native albumin (1 g/dL and 3.5 g/dL) to the recombinant receptor-binding region, RBD, of the SARS-CoV-2 spike protein contained in the S1 subunit. The spike protein S2 subunit failed to bind to 1 g/dL and 3.5 g/dL albumin.

As shown in Figure 4, native albumin binds the SARS-CoV-2 spike protein S1 subunit. The binding of the spike protein S1 subunit to native albumin was evidently higher to 3.5 g/dL, than to 1 g/dL native albumin (Figure 4). The spike protein receptor-binding domain (RBD) contained in the S1 subunit also bound to native albumin, and its binding was markedly enhanced by increasing native albumin concentrations (Figure 4).

The SARS-CoV-2 spike protein S2 subunit was not bound to 1 g/dL nor 3.5 g/dL native albumin. Indeed, the signal of the immunoprecipitation analysis was undetectable (Figure 4).

When 1 g/dL and 3.5 g/dL native albumin was replaced by 25% and 50% glycated albumin the binding of the SARS-CoV-2 spike S1 subunit was significantly reduced (Figure 4).

## 4. Discussion

The most relevant observation of this retrospective analysis was that in a large sample of 1555 confirmed COVID-19 patients from hospitals of the community of Madrid, on-admission albumin levels predicted <30 days in-hospital all-cause mortality. Moreover, native albumin bound the SARS-CoV-2 spike protein S1 subunit. This binding was more evident with 3.5 g/dL native albumin than with 1 g/dL. In addition, on-admission glucose levels above 125 mg/dL significantly increased <30 days in-hospital all-cause mortality for all albumin levels. Interestingly, glycated albumin reduced the ability of native albumin to bind the spike protein S1 subunit.

In the study, factors such as age, ICU admission and active cancer were associated to <30 days all-cause mortality in confirmed hospitalized COVID-19 patients. This fact was in the same line of evidence as previously reported findings [16,17,18]. In addition, as we also observed, independently of diabetes mellitus, high glucose levels on admission were also associated with a higher risk of mortality in COVID-19 patients, suggesting glucose levels are an independent risk factor for in-hospital mortality of COVID-19 patients [11]. Although an explanation for this observation is not the focus of the present study, a previously reported hypothesis has suggested that pancreatic beta-cells may be affected by COVID-19 reducing insulin secretion [19]. Moreover, the cytokines released during the COVID-19-induced inflammatory storm may favor insulin resistance, promoting an increase in glucose levels [19].

A few studies have associated hypoalbuminemia with higher mortality in COVID-19 patients [7,8,20]. In this regard, Huang et al., analyzed 299 adult COVID-19 patients, reporting hypoalbuminemia in almost 81% of non-survivors of severe COVID-19 patients [7]. Almost 15.4% of the studied patients (46/299) required ICU admission. Another study analyzed albumin levels in 134 patients [8]. Lower levels of both albumin and platelets were positively associated with the severity of COVID-19 pneumonia and death [8]. In the clinical setting, both studies had a limited impact, probably because of the limited number of patients, recruited in only a single institution, and it is important to point out that they were mainly carried out in critically ill COVID-19 patients [7,8].

A meta-analysis performed through 3 April 2020, included 11 articles with 910 patients, comparing albumin levels in severely and non-severely ill COVID-19 patients. The study did not specifically evaluate the association of albumin levels with mortality since a severe condition was defined as respiratory distress, ICU admission and/or death. However, it did show an association between hypoalbuminemia and severe COVID-19 [20]. Finally, in a small cohort of confirmed COVID-19 patients (*n* = 181) with radiographic evidence of pneumonia, Kheir et al. reported an association between albumin levels on admission and both outcome and mortality [21]. However, they did not find an association between albumin levels on admission and mortality, although this could be due to the small number of included patients (109 patients). In any case, taken together and from a clinical point of view, all these studies had limited attention. This could be related to different facts, for example only including patients from a single medical center. Moreover, there was high proportion of critically ill COVID-19 patients where it could be expected that albumin levels would correlate with worse disease progression.

The present study shows that low serum albumin levels on admission of confirmed COVID-19 infected patients were associated with a higher risk of all-cause mortality within 30 days of hospitalization. In fact, 88.9% of patients showing albumin levels ≥4 g/dL and 67.8% with albumin levels between 3.5 and 3.99 g/dL on admission were discharged alive. However, albumin levels below 2.5 g/dL were associated with an almost 60% higher <30 days in-hospital all-cause mortality.

The probability that albumin levels were affected by treatment should be discarded since albumin levels were always determined within 24h of hospital admission. The analysis showed only 11.2% of patients required ICU care and 2.8% developed deep venous thrombosis during hospitalization, indicating lesser severity of the COVID-19 disease. Therefore, our results suggest that on-admission albumin levels may be a predictor for all types of hospitalized COVID-19 patients, and that this was independent of other variables such as age, hypertension, dyslipidemia, active cancer, diabetes mellitus, COPD and glucose levels.

Initially, it is difficult to determine the possible mechanisms by which on-admission low levels of serum albumin may predict a higher in-hospital mortality risk. Albumin is synthesized in the liver and has a serum half-life of approximately 21 days. A meta-analysis suggested that COVID-19 patients with abnormal liver function had hypoalbuminemia [22]. Unfortunately, our database had no available data on liver function. However, due to the high number of COVID-19 patients showing albumin levels below 3.5 g/dL (597 patients), it is difficult to assume that all these patients had liver dysfunction. Acute inflammation is another important factor associated with low albumin [23]. Several COVID-19 patients suffer an excessive cytokine/chemokine response, known as “cytokine storm”. Therefore, it would be plausible to speculate that SARS-CoV-2 infection may be involved, at least in part, in decreased serum albumin. In this regard, a recent study has reported a significant inverse correlation between serum albumin and inflammatory indicators (hs-CRP) in COVID-19 patients, suggesting that hypoalbuminemia in SARS-CoV2 infection may be due to an inflammatory-mediated capillary leakage followed by a decreased albumin synthesis in hepatocytes [24,25]. In addition, there is an increased degradation and a decreased albumin synthesis during the inflammatory response due to cytokine-mediated reduced gene transcription, mainly mediated by Interleukin-6 [26]. However, C-reactive protein levels on admission were not associated with higher mortality, arguing against the possible involvement of inflammation on the mortalities associated with hypoalbuminemia. Other reports have shown an association between the temporal progression of C-reactive protein during hospitalization with in-hospital mortality. On admission, C-reactive protein levels have been rather associated with the extent of lung damage and the severity of illness in the early stages of COVID-19 [27,28].

As mentioned, albumin binds several molecules, as well as viruses. In the in vitro study, we demonstrated that native albumin binds the COVID-19 spike protein S1 subunit (the recombinant S1 subunit used contained the spike protein amino-acid sequence from 16 to 690 of the spike protein). Indeed, native albumin concentrations similar to those found in patients with normoalbuminemia (3.5 g/dL) markedly bound the COVID-19 spike protein S1 subunit, and this binding was drastically reduced by decreasing native albumin concentrations to 1 g/dL. An interesting observation, that evidently requires further investigation, was that the recombinant receptor-binding region, RBD, of the COVID-19 spike protein S1 subunit containing the S1 subunit amino-acid sequence from 319 to 541, also bound to native albumin. It is remarkable that the RBD region of the COVID-19 spike protein was used by the virus to interact with host angiotensin-converting enzyme 2 (ACE2) to infect human cells. The specificity of the interaction of native albumin with the SARS-CoV-2 spike protein was supported by the fact that the SARS-CoV-2 spike S2 subunit containing the amino-acid sequence from 697 to 1213 did not bind native albumin.

Any conformational change to albumin can affect its binding affinity to other molecules, including viruses. In this regard, albumin glycation alters the binding ability of albumin to other molecules [29,30]. Our in vitro analysis demonstrated that an increasing glycated albumin percentage in native albumin significantly reduced the ability of albumin to bind to the SARS-CoV-2 spike S1 subunit protein. Moreover, although the univariate and multivariate analysis showed that both albumin and glucose levels on admission were independent risk factors for in-hospital mortality, the fact that by increasing glucose values on admission, higher albumin values would be needed to be associated with lower mortality, could suggest the possibility of an interaction between both molecules affecting in-hospital survival of COVID-19 patients. Future studies are needed to further analyze this hypothesis.

It is recognized that treatment with albumin preparations or agents containing albumin may stimulate the immune response, even promoting the production of anti-albumin antibodies which might participate in some way in the elimination SARS-CoV-2 bound to albumin [31,32]. Therefore, one could speculate that native albumin could enhance the action of the immune system against the virus by binding SARS-CoV-2. Future studies are warranted to analyze this hypothesis.

## 5. Comments and Study Limitations

From the present results, many unresolved questions remain open. One of them is why serum albumin levels are reduced in some COVID-19-infected patients. Our study design made it impossible to draw any conclusion on whether hypoalbuminemia may be related to SARS-CoV-2. Interestingly, several works have reported a potential relationship between decreased serum albumin levels and COVID-19 infection, due to the inflammatory response in COVID-19 patients. It may be argued that hypoalbuminemia can only act as a biomarker for underlying disease severity, and not a direct pathogenic mediator involved in COVID-19 adverse outcomes or mortality. However, our in vitro findings revealed that the SARS-CoV-2 spike protein S1 subunit had the ability to bind albumin, which is used by virus to interact with host angiotensin-converting enzyme 2 (ACE2) to infect human cells and proliferate. Hence, it may be suggested that a direct pathogenic role of hypoalbuminemia in COVID-19 adverse events should not be ruled out. Additional studies are also needed to clarify whether increased mortality risk may also be related to the clinical condition due to hypoalbuminemia.

A study limitation is that the database contained limited information on the clinical characteristics of the patients. For example, it was not specified if there were any pregnant women, the disease severity grading on admission, clinical data such as oxygen saturation, pressure of oxygen, blood pressure or heart rate at admission, data about kidney and liver diseases, home medications, or in-hospital treatments. It is possible that some of these data may influence a patient´s albumin levels on admission. Nevertheless, independently of all these factors, albumin levels on admission were associated with all-cause <30 days in-hospital mortality.

Another limitation of the study is that albumin levels were only determined on admission. Albumin levels along the in-hospital stay of confirmed COVID-19 patients were not documented. Therefore, it was not possible to analyze if dynamic changes of albumin could improve its predictive value. Moreover, although glycated albumin reduced the ability of native album to bind the spike S1 subunit protein, future studies are needed to analyze the relationship between the level of glycated albumin and the in-hospital mortality of COVID-19 confirmed patients. Unfortunately, we have not had access to this data, since glycated albumin is not a parameter used in common clinical practice. In addition, it could have been more appropriate to use other indicators, such as HbA1c, since the blood glucose level at the time of hospital admission may have been affected by the treatment and diet before the hospital visits. Unfortunately, we have not had access to this data. However, in vitro studies revealed that SARS-CoV-2 replication and disease severity may be conditioned by intermittent increases in plasma glucose levels [33].

Therefore, summarizing, the present work shows the following features: (1) it was performed in a large number of patients including mainly hospitalized patients with less severe COVID-19 disease; (2) the patients came from several institutions; (3) the study analyzed the specific albumin intervals associated with higher and lower in-hospital mortality; (4) the study demonstrated the ability of native albumin to bind the SARS-CoV-2 spike S1 subunit protein and specifically the RBD spike region; (5) it was also postulated that glucose levels may influence this binding of albumin.

## 6. Conclusions

In conclusion, albumin levels on admission can predict <30 days in-hospital all-cause mortality independently of age, hypertension, dyslipidemia, diabetes mellitus, COPD, active cancer and glucose levels on hospital admission. Moreover, native albumin specifically binds the SARS-CoV-2 spike protein S1 subunit, but not the spike protein S2 subunit, apparently through the RBD region. Glycated albumin reduced the binding ability of the native albumin to the SARS-CoV-2 spike S1 subunit.

The search of prognostic biomarkers for COVID-19 disease has been a concern since the beginning of the pandemic [34]. In this regard, from a clinical point of view, the present results may suggest that, depending on the albumin levels on admission, a more aggressive therapy against COVID-19 may be considered at the beginning of hospitalization. These findings should be consolidated by expanding the study cohort in further studies.

## Figures and Tables

**Table 1:** Demographic characteristics in the multi-center cohort-study on the impact of admission serum albumin levels for the prediction of <30 days in-hospital all-cause mortality in COVID-19 patients, Spain, August–November 2020 (*n* = 1555).

Variables	Categories		COVID-19 Population *n* = 1555 *N* (%)
Age groups (years)	18–49		287 (18.5)
	50–69		494 (31.8)
	70–84		405 (26.0)
	≥85		369 (23.7)
Gender	Male		898 (57.7)
	Female		657 (42.3)
Diseases	Hypertension	No	767 (49.3)
		Yes	788 (50.7)
	Dyslipidemia	No	986 (63.4)
		Yes	569 (36.6)
	Diabetes mellitus	No	1157 (74.4)
		Yes	398 (25.6)
	Active cancer	No	1442 (92.7)
		Yes	113 (7.3)
	COPD	No	1400 (90.0)
		Yes	155 (10.0)
ICU admission	No		1381 (88.8)
	Yes		174 (11.2)
Outcome	Discharged		1052 (67.7)
	Death		503 (32.3)

Abbreviations—COPD: chronic obstructive pulmonary disease. ICU: intensive care unit.

**Table 2:** Mortality rate (%) according to demographic and clinical variables in the multi-center cohort-study on the impact of admission serum albumin levels for the prediction of <30 days in-hospital all-cause mortality in COVID-19 patients, Spain, August–November 2020 (*n* = 1555).

Variables		Categories	Death		*p* Value
			Not *n* = 1052 *N* (%)	Yes *n* = 503 *N* (%)	
Age groups (years)		18–49	272 (94.8)	15 (5.2)	<0.001
		50–69	424 (85.5)	70 (14.2)	
		70–84	228 (56.3)	177 (43.7)	
		≥85	128 (34.7)	241 (65.3)	
Gender		Male	598 (66.6)	300 (33.4)	0.296
		Female	454 (69.1)	203 (30.9)	
Diseases					
	Hypertension	No	595 (77.6)	172 (22.4)	<0.001
		Yes	457 (58.0)	331 (42.0)	
	Dyslipidemia	No	713 (72.3)	273 (27.7)	<0.001
		Yes	339 (59.6)	230 (40.4)	
	Diabetes mellitus	No	826 (71.4)	331 (28.6)	<0.001
		Yes	226 (56.8)	172 (43.2)	
	Active cancer	No	1009 (70.0)	433 (30.0)	<0.001
		Yes	43 (38.1)	70 (61.9)	
	COPD	No	979 (69.9)	421 (30.1)	<0.001
		Yes	73 (47.1)	82 (52.9)	
ICU admission		No	975 (70.6)	46 (29.4)	<0.001
		Yes	77 (44.3)	97 (55.7)	
at Admission Parameters					
	Albumin (g/dL)	<3.5	304 (50.9)	293 (49.1)	<0.001
		≥3.5	748 (78.1)	210 (21.9)	
	Glucose (mg/dL)	<100	232 (74.8)	78 (25.2)	<0.001
		100–125	387 (72.9)	144 (27.1)	
		>125	363 (57.0)	274 (43.0)	
	C-Reactive Protein (mg/dL)	≤0.5	30 (71.4)	12 (28.6)	0.391
		>0.5	439 (64.9)	237 (35.1)	

Abbreviations—COPD: chronic obstructive pulmonary disease. ICU: intensive care unit.

**Table 3:** Univariate and multivariate analysis showing mortality odds ratios (OR) adjusted by age and by all the included variables in the multi-center cohort-study on the impact of admission serum albumin levels for the prediction of <30 days in-hospital all-cause mortality in COVID-19 patients, Spain, August–November 2020 (*n* = 1555).

Variables	Categories	Crude		Age Adjusted		Full Adjusted *	
		OR (95%CI)	*p* Value	OR (95%CI)	*p* Value	OR (95%CI)	*p* Value
Age groups	18–49	1		NA	NA	1	
	50–69	2.99 (1.68–5.34)	<0.001			1.85 (0.97–3.52)	0.060
	70–84	14.07 (8.07–24.53)	<0.001			11.70 (6.09–22.52)	<0.001
	≥85	34.14 (19.45–59.90)	<0.001			40.63 (20.86–79.15)	<0.001
Gender	Male	1	0.296	1	<0.001	1	
	Female	0.891 (0.72–1.11)		0.618 (0.48–0.80)		0.74 (0.56–0.99)	0.041
Active Cancer	No	1		1		1	
	Yes	3.79 (2.55–5.64)	<0.001	3.33 (2.13–5.21)	<0.001	3.87 (2.34–6.42)	<0.001
Dyslipidemia	No	1		1		1	
	Yes	1.77 (1.42–2.20)	<0.001	1.07 (0.83–1.37)	0.601	1.06 (0.79–1.43)	0.688
Diabetes	No	1		1		1	
	Yes	1.90 (1.50–2.40)	<0.001	1.20 (0.92–1.57)	0.179	1.03 (0.75–1.42)	0.866
Hypertension	No	1		1		1	
	Yes	2.50 (2.00–3.12)	<0.001	0.79 (0.60–1.04)	0.100	0.86 (0.62–1.19)	0.368
COPD	No	1		1		1	
	Yes	2.61 (1.87–3.65)	<0.001	1.46 (1.01–2.10)	0.043	1.02 (0.67–1.54)	0.930
ICU admissions	No	1		1		1	
	Yes	3.03 (2.20–4.17)	<0.001	8.96 (5.94–13.51)	<0.001	8.76 (5.68–13.50)	<0.001
Albumin (g/dL)	<3.5	1		1		1	
	≥3.5	0.29 (0.23–0.36)	<0.001	0.44 (0.34–0.56)	<0.001	0.48 (0.36–0.62)	<0.001
Glucose (mg/dL)	<100	1		1		1	
	100–125	1.11 (0.80–1.52)	0.535	1.00 (0.68–1.45)	0.990	1.02 (0.69–1.51)	0.937
	>125	2.24 (1.66–3.03)	<0.001	1.63 (1.15–2.32)	0.006	1.56 (1.07–2.29)	0.022

* Adjusted by all variables included in the table. Abbreviations—COPD: chronic obstructive pulmonary disease. ICU: intensive care unit. NA: not applicable. The value of the Hosmer–Lemenshow goodness-of-fit statistic was 12.59 and 0.127 the corresponding *p*-value. This indicates that the proposed model seems to fit quite well.

## Data Availability

Anonymized database based on data of 24 different public hospitals of the Community of Madrid was provided by the Madrid Community Ministry of Health. However, we have not permission to share database. Consequently, we cannot upload the databases to any public repository.

## Supplementary Materials

The following supporting information can be downloaded at: https://www.mdpi.com/article/10.3390/jcm11030587/s1. Table S1: sociodemographic and health variables. Sociodemographic and health variables of referral (*n* = 801) and validation (*n* = 754) groups to prove that native albumin data can be used to predict outcomes of COVID-19 patients. Table S2: Multivariate analysis mortality Odds Ratis (OR) adjusted by all included variables. Validation of the proposed prediction model.
