# Supplementary material for: Albumin Binds COVID-19 Spike 1 Subunit and Predicts In-Hospital Survival of Infected Patients—Possible Alteration by Glucose
Source: J Clin Med. 2022 Jan 25;11(3):587. doi: 10.3390/jcm11030587 (PMC8836760; doi:10.3390/jcm11030587)
Supplement: Supplementary file 1 [file jcm-11-00587-s001.zip › jcm-1523551-supplementary.pdf]

Supplementary Table S1. Sociodemographic and health variables.

VARIABLES	CATEGORIES	REFERRAL GROUP (N=801) N (%)	VALIDATION GROUP (N=754) N (%)	P-Value
Age groups (years)	18–49	140 (17.5)	147 (19.5)	0.099
	50–69	277 (34.6)	217 (28.8)	
	70–84	204 (25.5)	201 (26.7)	
	≥85	180 (22.5)	189 (25.1)	
Gender	Male	460 (57.4)	438 (58.1)	0.792
	Female	341 (42.6)	316 (41.9)	
DISEASES				
Hypertension	No	400 (49.9)	367 (48.7)	0.618
	Yes	401 (50.1)	387 (51.3)	
Dyslipidemia	No	509 (63.5)	477 (63.3)	0.908
	Yes	292 (36.5)	277 (36.7)	
Diabetes mellitus	No	605 (75.5)	552 (73.2)	0.295
	Yes	196 (24.5)	202 (26.8)	
Active cancer	No	742 (92.6)	700 (92.8)	0.877
	Yes	59 (7.4)	54 (7.2)	
COPD	No	719 (89.8)	681 (90.3)	0.715
	Yes	82 (10.2)	73 (9.7)	
ICU admission	No	711 (88.8)	670 (88.9)	0.952
	Yes	90 (11.2)	84 (11.1)	
Outcome	Discharged	556 (69.4)	496 (65.8)	0.126
	Death	245 (30.6)	258 (34.2)	

Abbreviations. - COPD: Chronic Obstructive Pulmonary Disease. ICU: intensive care unit

Supplementary Table S2. Multivariate analysis showing mortality Odds Ratios (OR) adjusted by all included variables.

VARIABLES	CATEGORIES	REFERRAL GROUP OR (95%CI)	VALIDATION GROUP OR (95%CI)
Age groups (years)	18–49	1	1
	50–69	2.42 (0.99–5.90)	1.18 (0.45–3.08)
	70–84	9.33 (3.70–23.55)	15.41 (6.00–39.61)
	≥85	33.97 (13.26–87.01)	56.41 (21.44–148.42)
Gender	Male	1	1
	Female	0.73 (0.50–1.08)	0.82 (0.54–1.24)
DISEASES			
Hypertension	No	1	1
	Yes	0.98 (0.64–1.51)	0.70 (0.42–1.17)
Dyslipidemia	No	1	1
	Yes	1.10 (0.73–1.64)	1.00 (o.63–1.58)
Diabetes mellitus	No	1	1
	Yes	1.29 (0.83–1.99)	0.78 (0.47–1.28)
Active cancer	No	1	1
	Yes	2.74 (1.43–5.25)	7.28 (3.08–17.24)
COPD	No	1	1
	Yes	1.09 (0.71–1.62)	1.17 (0.76–1.69)
ICU admission	No	1	1
	Yes	6.70 (3.78–11.87)	13.02 (6.48–26.16)
Albumin (g/dL)	<3.5	1	1
	≥3.5	0.56 (0.39–0.82)	0.37 (0.25–0.56)
Glucose (mg/dL)	<100	1	1
	100–125	0.95 (0.54–1.64)	1.12 (0.63–1.98)
	>125	1.43 (0.84–2.43)	1.94 (1.09–3.45)

Abbreviations. - COPD: Chronic Obstructive Pulmonary Disease. ICU: intensive care unit. "The value of the Hosmer-Lemenshow goodness-of-fit statistic was 6.06, 10.99 (referral, validation) and the corresponding p-values 0.640,0.203. This indicates that the models seem to fit quite well."
